# Health Condition Estimation of Bearings with Multiple Faults by a Composite Learning-Based Approach

**DOI:** 10.3390/s21134424

**Published:** 2021-06-28

**Authors:** Udeme Inyang, Ivan Petrunin, Ian Jennions

**Affiliations:** 1 Integrated Vehicle Health Management Centre, Cranfield University, Cranfield MK43 0AL, UK; i.jennions@cranfield.ac.uk; 2 Centre for Autonomous and Cyberphysical Systems, Cranfield University, Cranfield MK43 0AL, UK; i.petrunin@cranfield.ac.uk

**Keywords:** multiple faults, diagnostics, complementary, deep learning, health management

## Abstract

Bearings are critical components found in most rotating machinery; their health condition is of immense importance to many industries. The varied conditions and environments in which bearings operate make them prone to single and multiple faults. Widespread interest in the improvements of single fault diagnosis meant limited attention was spent on multiple fault diagnosis. However, multiple fault diagnosis poses extra challenges due to the submergence of the weak fault by the strong fault, presence of non-Gaussian noise, coupling of the frequency components, etc. A number of existing convolutional neural network models operate on a distinct feature that is not enough to assure reliable results in the presence of these challenges. In this paper, extended feature sets in three homogenous deep learning models are used for multiple fault diagnosis. This ensures a measure of diversity is introduced to the health management dataset to obtain complementary solutions from the models. The outputs of the models are fused through blending ensemble learning. Experiments using vibration datasets based on bearing multiple faults show an accuracy of 98.54%, with an improvement of 2.74% in the overall effectiveness over the single models. Compared with other technologies, the results show that this approach provides an improved generalized diagnostic capability.

## 1. Introduction

Rolling bearings are used in a sizable number of machines to support and allow relative motion between machine parts that are in contact. They are found in operation in various industrial environments and are subjected to varied load conditions/speeds over a long time. The tough environment in which bearings operate, poor lubrication, manufacturing or installation errors promote single and multiple faults. Faults in bearings can bring about downtime, large financial losses, and in some cases death, due to abrupt failure while in operation [1,2,3]. To reduce or eliminate losses, accurate and reliable diagnosis is of utmost importance.

Different approaches have been proposed for the diagnosis and prognosis of bearings. These include model-based, data-driven and signal processing-based, knowledge-based, active fault diagnosis, and hybrid approaches [4]. The model-based approaches are formed from a physical representation of the process using mathematical equations [5] as well as prior physical knowledge of the system. However, these types of models are more demanding to build. The data-driven approach is an approach where fault information is obtained from data collected from the system. The hybrid approach is a combination of any of the above analytical redundancy-based diagnosis [5,6,7].

A huge amount of data, from which important health information of rotating machines can be extracted, are now readily available. This means researchers and industries can easily rely on data-driven approaches to ascertain the status of machines [6,8]. However, the effective deployment of this approach requires careful consideration of the conditions to which a bearing is subjected. Some of these conditions include varied load, fluctuation in speed, the presence of non-informative impulsive noise in the vibration signal, varied signal-to-noise ratios, the presence of single and/or multiple faults in the vibration signal. If the goals of predictive maintenance are to be preserved, it is necessary for the utilized system to be flexible. Flexibility, in this context, is the ability of the diagnostic system to give satisfactory results in a multitude of conditions/components. Therefore, a flexible data-driven system should give reliable results for single and multiple faults.

Vibration signal analysis involves separating the collected vibration signal from the fault characteristic signal and identifying the fault in the system by analyzing the separated signal [9]. In the last few decades, many signal-based fault diagnosis methods have been developed for fault feature identification [4]. The spectral kurtosis has been deployed in fault identification based on its ability to provide an indication of the variation of impulsiveness of a signal with frequency [10,11]. It has been useful in providing the optimum frequency band for demodulation. Considering the bearing fault signal as cyclostationary, cyclic spectral analysis [12] has been exploited in bearing fault detection. This method has recorded some good results in bearing fault detection, even under a strong masking signal, by utilizing the correlation statistics between spectral components spaced apart by some frequency shift [13]. Bicoherence analysis, on the other hand, relies on non-linear simultaneous interaction and quadratic phase coupling between the frequency components in a bearing signal for fault detection. It has also been effective for bearing fault detection, even in conditions of low signal to noise ratios [14].

However, spectral kurtosis, bicoherence analysis and cyclic spectral coherence, when used in isolation, do not give reliable results in some scenarios. For instance, a kurtogram can be used to select the frequency band with the highest spectral kurtosis value for demodulation. When this technique is employed in industrial applications where impulsive noise is present, spectral kurtosis may select a source different from the damage. This is because many frequency bands may have been excited simultaneously. In transient vibratory signals, the bicoherence would only give limited diagnostic efficacy [15]. Even with a change in the excitation frequency range, the cyclic spectral coherence will not change significantly. This makes cyclic spectral coherence more suitable for early fault detection [16]. Other studies, such as Gao et al. [4], have highlighted the weakness of some signal-based health indices extraction methods. These limitations are because signal processing transforms are mostly complementary, and not independent [17]. Complementarity requires that the signal processing techniques can account for each other’s deficiency when solving the same problem [18]. It is vital to the success of a flexible diagnostic system.

In collaboration with machine learning algorithms, including deep learning algorithms, signal processing techniques have been used to improve the fault diagnosis process. Deep learning (DL) algorithms can solve the difficult and challenging task of feature extraction and feature selection [19]. DL methods, such as the convolutional neural network (CNN), autoencoder, deep belief network (DBN), recurrent neural network, and generative adversarial network, perform the tasks of feature extraction and selection independently.

Using a 1D CNN directly on 1D vibrational data would not provide good results, as 1D CNN suffers from shift-variant problems [20]. Therefore, to achieve better results, the vibration data were converted into 2D representation for use with a 2D CNN. This creates a feature space which allows for the learning of complex patterns from this space directly without resorting to manual feature engineering [21]. Verstraete et al. [22] relied on representative images of the time-frequency domain presentation of vibration data input to a deep convolutional neural network for diagnosis of bearing. They compared the accuracy of three time-frequency analysis techniques: Short-Time Fourier Transform (STFT), Continuous Wavelet Transform (CWT), and Hilbert–Huang Transform (HHT) on their CNN architecture. Similarly, Wan et al. [23] compared the performance of different inputs in their research by using eight distinct types of inputs namely STFT, Constant-Q Gabor Transform, Instantaneous Frequency, Fast Kurtogram, HHT, Wigner–Ville Distribution, Fourier Synchro-squeezed Transform (FST), and CWT. Other academics have used statistical features for rotating machinery diagnostic systems [24,25,26,27,28]. Although the performance of fault diagnosis systems depends on the feature spaces used [28], it was observed that these feature spaces were used arbitrarily and not combined in such a way to achieve generalization between different conditions, that the bearings may be subjected. For instance, in [9] while the FFT would give unsatisfactory results with a signal whose frequency components changes with time, the wavelet transform is known to suffer from fixed scale resolution which would affect its real-life applications, and the HHT suffers from instability in its signal decomposition process.

Ensemble learning can improve the performance of a diagnostic system by overcoming some challenges of the single learner. This is achieved by exploiting the gains of diversity between base-learners. Ensemble learning has proven to be a successful approach, which has found application in condition monitoring. Ensemble model creation involves building the ensemble and combining the ensemble members [29]. Diversity between the ensemble members can be obtained by using different topologies in the constituent learners, varying the algorithm of the base-learners, and varying the dataset. Boosting, bagging, the use of different data sources, and the use of different preprocessing techniques are some of the methods used to introduce variation to the dataset. Liang et al. [30] proposed the training of a few DBNs based on different hyper-parameters to form an improved ensemble learning for bearing diagnosis. Shao et al. [31] constructed an ensemble of deep autoencoders, where raw vibration data were fed to 15 deep autoencoders with different activation functions for bearing classification. Li et al. [32] assembled three diverse types of autoencoders (denoising autoencoder, sparse autoencoder, and sparse autoencoder with linear decoder), using inputs from raw bearing vibration data randomly obtained through bootstrapping. Han et al. [33], proposed a dynamic ensemble of a CNN based on wavelet packet transform for rotating machine diagnosis. Ma et al. [34] utilized FFT input in an ensemble of a CNN, DBN and stacked autoencoders for rotor bearing diagnosis. In all of these, one preprocessing technique was used.

In the current work, a novel aspect is that an extended feature set is used in multiple fault diagnosis. Our contribution therefore includes the proposition of a diagnostic model that relies on complementary transforms in an ensemble for multiple bearing fault diagnosis. Three preprocessed approaches are applied on the vibration signal to obtain bicoherence maps, cyclic spectral coherence maps, and the kurtogram. These inputs are fed to deep learning-based models under different working conditions in a blending ensemble architecture. The remainder of this article is organized as: Section 2 describes the materials and methods, signal processing techniques used for preprocessing, and ensemble learning/the specific ensemble learning approach. The results are provided in Section 3. Section 4 deals with the discussion, while Section 5 highlights the conclusions.

## 2. Materials and Methods

### 2.1. Convolutional Neural Network

A CNN is a supervised, feed-forward deep-learning model designed for spatial hierarchies’ automatic learning of low and high-level features. The CNN has proved successful in many applications, such as video and image recognition, bioinformatics, natural language processing. A typical CNN architecture is made up of the convolution layer, pooling layer, and the fully connected layer, from which its operations are carried out.

The convolutional layer is a key layer that performs the feature extraction to obtain the feature maps. The convolution operation is performed in this layer. It is a mathematical operation in which the convolution kernel is applied to the input to produce an output known as a feature map. Before training the CNN, the padding, stride, the number of the kernels, and the size of the kernels are defined. A feature map is obtained through a sum operation of all the results of the element-to-element products between the input tensor and the specified filter. This process is repeated through the application of a different number of filters and sizes to obtain a varied number and depth of feature maps that describe the characteristics of the input tensor [35]. Appendages of a row and a column of zeros through zero paddings are made on the input to maintain the feature maps’ in-plane dimension and allow the application of more layers. A filter bank is used to connect each unit of the feature map from the convolution layer to the previous layers’ feature map. In summary, the convolutional layer computes the dot product of the input $X_{q-1}^{\left(i\right)}$ of the convolution channel (by convolving) with the filter weight matrix $W_{q}^{\left(i,m\right)}$ with a weighted bias matrix $B_{q}^{\left(m\right)}$ added and passed through an activation function such as the Rectified Linear function. This operation is shown in Equation (1). The ReLU is widely used because of its advantages of overcoming gradient vanishing problems encountered in the backpropagation stage of neural network training and requiring reduced calculation [36]. Other activation functions previously in use were tangent function and logistic function.

(1) Xqm=ReL∪(∑i=1IWq(i,m)×Xq−1(i)+Bq(m)),

To reduce the number of subsequent learnable parameters and, therefore, the computational complexity, the down-sampling operation is performed by the pooling layer. The pooling layers are found between successive convolutional layers. They are used to reduce the spatial representation of the data, thereby controlling overfitting [37]. Mathematically, the pooling operation can be represented by Equation (2) below, where $\beta_{q-1\;}^{\left(m\right)}$ is the multiplicative bias, and $b_{q}^{\left(m\right)}$ is the additive bias.

(2) $$X_{q}^{m}=f\left(\beta_{q-1}^{\left(m\right)}down\left(X_{q-1}^{\left(m-1\right)}\right)+b_{q}^{\left(m\right)}\right),$$

The inputs to the pooling layer are divided into disjointed regions with the dimension [M × N], where “M” is the number of mini-batches and “N”, the maximum (max) or mean feature activations as the case may be, is used to obtain the “pooled” convolved features over these regions [38].

The third key layer is the fully connected layer. The higher-order features produced in the previous layers are used to create class probabilities, also known as scores.

### 2.2. Blending Ensemble Learning

Building an ensemble model involves choosing a suitable method for training the accurate diverse models and selecting a suitable way of combining the output of the base inducers. The base inducers can be combined using methods such as stack generalization/blending [39], using different algebraic functions [40], non-linear combination methods (for instance, Dempster–Shafer belief methods) [41]. Stacked generalization is an ensemble learning approach that applies a meta-learner and out-of-fold prediction of the training set to detect the best way of combining the base models’ outputs [42]. A variant of stacked generalization is blending. Blending reduces information leaks and it is more straightforward [42]. In blending, the predictions from each of the tier-zero models are fed as training data to the meta-learner. The results are obtained from the predictions of the meta-learner. The proposed approach in this article utilizes blending ensemble learning. Different preprocessing techniques were used in creating the ensemble members.

The meta-learner in the proposed method is a multiclass Support Vector Machine (SVM). SVMs are primarily two class machine learning algorithms that can be used for solving classification and regression problems. The SVM works by creating an optimal hyperplane separation between the datasets, with the minimum distance between the datapoints described as support vector. A constrained quadratic optimization is solved to achieve this objective by using the structural risk minimization. In practice, a multiclass SVM can be built using different techniques such as one versus one, one versus all, and a directed acrylic graph. However, the one versus one coding design for seven classes was applied here. In this approach, the multiclass classification task is reduced to a multiple binary classification problem, and each hyperplane is constructed from training samples of two classes chosen from total “K” classes. The Error-Correcting Output Code (ECOC) in MATLAB [43] for multiclass learning was implemented in this study.

### 2.3. Signal Processing Techniques

#### 2.3.1. Spectral Kurtosis and Kurtogram

R. F. Dwyer proposed kurtosis as a mathematical tool for determining in the frequency domain, the presence and location of non-Gaussian components in a signal. Using the Wold–Cramer decomposition, Antoni [44] described the output of a causal, linear, and time-varying system as being a nonstationary stochastic process.

(3) $$Y\left(t\right)=\int_{-\infty }^{+\infty }e^{j2\pi ft}H\left(t,f\right)dX\left(f\right),$$

where “*H(t, f)*” represents the time-varying function interpreted as the complex envelope of the process “*Y(t)*” at a frequency “*f*”, while “*dX(f)*” stands for the spectral process associated with the process. The key assumption on which spectral kurtosis is applied is the conditional non-stationarity (CNS) of the process under consideration. Hence, the energy-normalized fourth-order cumulant of the CNS process will give the measure of the peakedness of the probability density function of the process at frequency “*f*”. Hence, the spectral kurtosis is defined as:

(4) $$Sk_{Y}\left(f\right)=\frac{s_{4Y}}{s_{2Y}^{2}}-2,\;f\neq 0,$$

where the second-order instantaneous moment *S_2NY_*(*f*) to estimate the strength of the energy of the complex envelope of the process at frequency *“f”* is given by:

(5) $$S_{2NY}\left(f\right)=\frac{E\left\{{\left|H\left(t,f\right)dX\left(f\right)\right|}^{2n}|\omega \right\}}{df}=E\left\{{\left|H\left(t,f\right)\right|}^{2n}\right\}\cdot S_{2nX},$$

The spectral cumulant with order *2n* ≥ 4 has a property that is zero for Gaussian random processes. The spectral kurtosis for a CNS can be estimated by:

(6) $$SK_{V}\left(f\right)=\frac{SK_{Y}}{{\left[1+\rho \left(f\right)\right]}^{2}}f\neq 0,$$

where in Equation (6), *ρ(f) = S_2N_(f)/S_2Y_(f)* is the noise to signal ratio. It can be observed that when the value *ρ(f)* is low, *SK_v_* is equal to *SK_Y_*. Hence, the concept behind the spectral kurtosis is to have a quantity that outputs zero values when the signal is Gaussian but gives high values when the signal of interest is transient. Antoni et al. [45] introduced spectral kurtosis for the analysis of rotating machine signals using some quasi-analytic filter banks. Here, the hidden non-stationarity of a particular frequency band is obtained by calculating the kurtosis value. The kurtogram gives an optimum combination of a frequency/frequency resolution. The limits of the kurtogram level are based on the length “*L*” of the signal “*Y(t)*”, which is obtained using *Log_2_(L)* − 7.

#### 2.3.2. Bicoherence

Rotating machine faults can be related to the nonlinearity occurring in the machine itself. Building from the deficiency of power spectrum in that, phase information is lost during the power spectral analysis, the bispectrum analysis was introduced. Bispectrum analysis is one of the Higher-Order Spectra (HOS) or polyspectra analysis techniques and can be described as a double Fourier transform of the third-order moment (skewness) of a signal. It gives a decomposition of a signal’s skewness over frequency, thereby identifying the distribution and magnitude of nonlinear coupling between frequencies in the signal. The bispectrum analysis gives information about the non-Gaussianity of a signal. This is based on the principle that if a Gaussian input is fed to a linear system, the output of such a system will be Gaussian. HOS, in this case, will give no information. However, when a Gaussian input is fed to a non-linear system, the output will be non-Gaussian. Bispectrum can be computed using the direct or the indirect methods. For a vibration signal given by *y(t)*, the bispectrum can be calculated using Equation (7) below, as:

(7) $$B\left(f_{1}\;,f_{2}\;\right)=\underset{T\;\to \infty }{\lim}\frac{1}{T}E\left[Y\left(f_{1}\;\right)Y\left(f_{2}\;\right)Y*\;\left(f_{1}+f_{2}\;\right)\;\right],\;$$

where “*Y(f)*” represents the discrete Fourier transform of the vibration signal, “*E*[.]” is the expectation operation or statistical average of the ensemble, “*” represents the complex conjugate, and “*f1*” and “*f2*” are independent frequencies and “*T*” is the duration of the signal [46].

The bispectrum estimate depends on the energy of the signal at the bifrequency. Hence, at bifrequency where the energy is low, the variance of the bispectrum will be lower, and vice versa for bifrequency with high energy. A common way to resolve this undesirable property in the bispectral estimate is to normalize the bispectrum to obtain an approximately flat variance across all bifrequency. The result is known as the bicoherence spectrum, and can be expressed as shown in Equation (8).

(8) $$b^{2}\left(f_{1},f_{2}\right)=\frac{{\left|B\left(f_{1},f_{2}\right)\right|}^{2}}{E\left[{\left|Y\left(f_{1}\right)\;Y\left(f_{2}\right)\right|}^{2}\right]\;E\left[{\left|Y(f_{1}+f_{2})\right|}^{2}\right]}$$

#### 2.3.3. Cyclic Spectral Coherence

Cyclo-stationary processes, or periodically correlated processes, are stochastic processes that exhibit some hidden periodicity. A typical cyclo-stationary process occurs in rotating machinery, such as rolling bearings, when faults occur on them. The impacts occurring several times are produced by these faults and are modulated by the shaft rotating frequency [47]. This property can be used to detect faults in the rotating machine. An *n*th order cyclo-stationary signal is said to be a signal “*y(t)*” whose *n*th-order statistic is periodic. The first-order cyclo-stationary signal (CS1) is represented in Equation (9), where *M_y_*, the statistical mean, is periodic with the period *T* of the signal *y(t)*, while “*E*” is the ensemble average. Martin et al. [48], described them as signals with finite-amplitude additive periodic components and consequently, they exhibit lines in their power spectral density.

(9) $$M_{y}\left(t\right)=M_{y}\left(t+T\right)=E\left\{y\left(t\right)\right\},$$

The first-order cyclo-stationary signal is mostly generated by processes such as imbalance, misalignments, and components such as flexible couplings [49]. However, for second-order cyclo-stationary signals (CS2), the autocorrelation function, which is periodic with time, can be calculated using:

(10) $$R_{yy\left(t,ꞇ\right)}=R_{yy}\left(t+T,ꞇ\;\right)=E\left\{y\left(t\;-\frac{ꞇ}{2}\right)y\left(t+\frac{ꞇ}{2}\right)\right\}$$

where the time lag is represented by “*ꞇ*”. Second-order cyclo-stationary signals are prevalent in rotating machines’ vibration. These vibrations are stochastic processes with a periodic amplitude and/or frequency modulation.

When a two-dimensional Fourier transform is performed on the autocorrelation function, the spectral correlation is obtained. A tool designed to describe the CS1 and CS2 in the frequency–frequency domain is the cyclic spectral correlation, defined by Equation (11).

(11) $$CScor\left(\alpha ,f\right)=\underset{n\to \infty }{\lim}\frac{1}{W}E\left\{Y{\left(f\right)}_{d}\left[y\left(t\right)\right]\;\;Y{\left(f\right)}_{d}\left[y\left(t+ꞇ\right)\right]*\right\}\;$$

where “*f*” represents the spectral frequency of the carrier signal, α is the cyclic frequency or modulation frequency, and “*Y(f)*” is the Fourier transform of the signal of duration “*d*”. Thus, for a wave signal given by “*Y*”, the spectral correlation can be described as displaying the strength of “*Y*” that is carried and modulated at all combinations (α, *f*) [50]. A normalization term can be added to Equation (12) to obtain the cyclic spectral coherence, which is highly effective in detecting rotating machine faults:

(12) $$CScoh\left(\alpha ,f\right)=\frac{CScor\left(\alpha ,f\right)}{\sqrt{CScor\left(0,f\right)CScor\left(0,f-\alpha \right)}}$$

### 2.4. Structure of the Proposed Method

**Figure 1:**
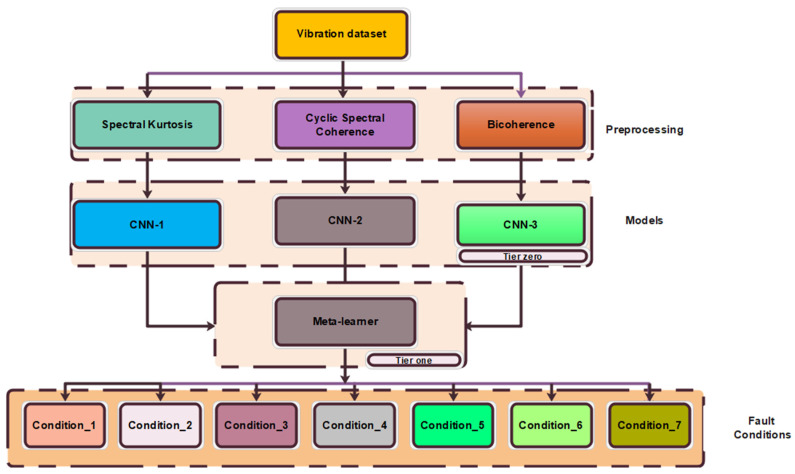
Proposed architecture for multiple faults of bearings.

The proposed method is shown in Figure 1. In this approach, the ensemble building step involved the use of three carefully chosen preprocessing techniques namely: cyclic spectral coherence, spectral kurtosis, and bicoherence on the vibration signal. These base-learners were combined using blending ensemble learning strategy. The entire method is described in the following simplified steps.

Step 1. The vibration signal is preprocessed using complementary signal processing techniques.
Step 2. Divide the data into training, validation, and testing sets.
Step 3. Choose hyperparameters and train the tier zero models.
Step 4. Obtain predictions from the tier zero models using the validation set.
Step 5. Train the meta-learner with predictions from tier zero models.
Step 6. Estimate the health conditions of the bearings using the testing set.

## 3. Experiments

### 3.1. Dataset Description

**Figure 2:**
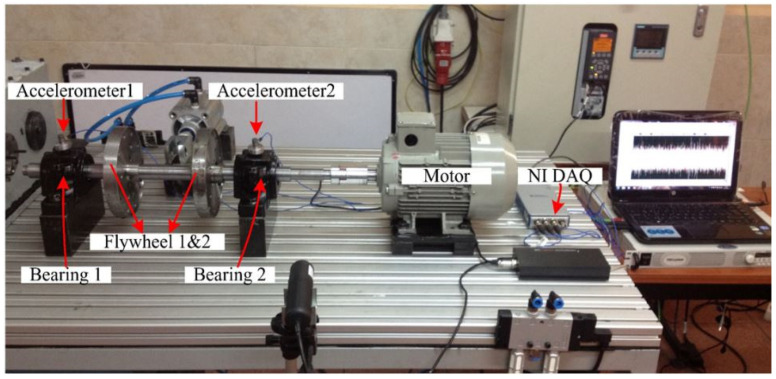
Universidad Politécnica Salesiana test rig [52].

The performance of the proposed approach to multiple faults of the rolling bearing are tested on the experimental dataset obtained from the test rig of Universidad Politécnica Salesiana Ecuador [51], shown in Figure 2. The experimental setup consists of a 30 mm diameter shaft on which two rolling element bearings are mounted. This set-up is driven by an inverter-controlled motor. When loads (L2 and L3) are required in the system, they are introduced using flywheels (F2 and F3). Condition L1 signifies a scenario where no flywheel or zero loads was introduced to the system. L2 and L3 represent other loading conditions, in which two and three flywheels, respectively, were used in the setup. The vibration dataset was acquired at three different rotational speeds of 8 Hz, 10 Hz and 15 Hz.

Accelerometers are installed on the housing of the bearings, as shown in Figure 2. Each MATLAB structure of the vibration dataset is made up of five fields, which includes: accelerometer one readings, accelerometer two readings, a sampling rate of 50 kHz, the shaft rotating speed, and sampling time. The total time duration of each of the signals is 20 s. Using these conditions, the experiment was repeated over five (5) runs. Hence, seven (7) fault classes of the bearing were obtained. For this study, two key assumptions are made: (1) the distance between bearing 1 and bearing 2 can be smaller but not larger than that used in this experimental setup. (2) the reverse combination of these faults were not considered. (3) Readings from accelerometer 1 alone under the influence of bearing 2 were used. A summary of these fault classes is presented in Table 1.

### 3.2. Data Preprocessing

Three signal processing techniques, namely: cyclic spectral coherence, bicoherence, and spectral kurtosis, have been chosen to leverage on their complementarity to diagnose bearing multiple faults. In the data preparation stage of the bicoherence maps, a key consideration is to choose a data segment that is long enough to create an asymptotically unbiased and stable estimation, while also having a good frequency resolution [53]. In this paper, each bicoherence map was created using a frame size of 0.8 s, with the Number of Fast Fourier Transform (NFFT) length being 512, Hanning window applied to each of the 200 data segments, using a percentage overlap of 60. Hence, for each of the fault classes listed in Table 1, 225 samples are obtained for each of the signals and a total of 450 samples from two runs of the experimental set up.

To achieve good accuracy with the cyclic spectral coherence-based convolutional neural network, a compromise must be made between the resolution of the cyclic spectral coherence maps and the computational cost [21]. Hence, a frame size of 0.8 s (40,000 data points) and the highest cyclic frequency to be scanned as 300 Hz was used to create each of the 450 cyclic spectral coherence maps. A lower sampling point will ensure that the computation time is less; however, the accuracy of the model will be drastically reduced.

The spectral kurtosis was another technique used to preprocess the vibration signal. Udmale et al. [54] confirmed that the maximum decomposition level of the kurtogram revealed more frequency information, because the plane (f, ∆f) becomes finer with an increase in the decomposition level. However, this maximum level of decomposition is determined by the length of the signal used. In this paper, a frame size of 0.8 s with a maximum decomposition level of 8 was used to create 450 kurtogram from two runs of the machine. Irrespective of the preprocessing technique deployed, the entire images were 224 × 224 pixels.

### 3.3. Training, Validation, and Testing Sets

The dataset was divided into training, validation, and testing sets. The test set was obtained from run 3 of the machines while the training and validation sets were drawn from run 1 and run 2 of the machines. Table 2 shows the composition of the training, validation and test sets used. One of the challenges of a small sample training size is overfitting. This problem can be overcome using a combination of methods, including data augmentation, to ensure more diversity [55] of the training dataset. Rotation, horizontal and vertical translation data augmentation techniques were implemented on the training dataset. The rotation augmentations were carried out by safely rotating the images on an axis between a range of −15° and 15°. Bias in position was tackled by introducing a range of random horizontal and vertical translations of −3 and up to 3 pixels. CNN-1, CNN-2, and CNN-3 models were trained on generically preprocessed inputs based on spectral kurtosis, cyclic spectral coherence, and bicoherence using MATLAB with learning rates of 0.0005, 0.0005, and 0.001, respectively. The structure of the tier-zero models is listed in Table 3.

### 3.4. Network Outcomes

Most often, an empirical comparison is carried out by applying algorithms on various datasets and evaluating the performance of the classifiers that the algorithm(s) have produced [56]. Hence, to fully evaluate the efficiency of the individual models and that of the ensemble model, different performance metrics were used. These metrics are briefly defined here as:

1. Overall accuracy: This is a metric that gives the overall effectiveness of a classifier. Accuracy is given by Equation (13):
  (13) $$Overall\;accuracy=\frac{Correct\;Predictions}{Total\;Predictions}\times 100,$$
2. Recall: This performance metric estimates the probability of a classifier to identify positive labels. Recall is also known as sensitivity or true positive rate.
  (14) $$Recall=\frac{True\;Positives}{True\;Positives+False\;Negatives}\times 100,$$
3. Precision: This is the ratio of correctly classified positive samples to the number of samples which the network labels as positive. This metric is also referred to as the positive predictive value of the network. It is mathematically given in Equation (15):
  (15) $$Precision=\frac{True\;Positives}{True\;Positives+False\;Positives}\times 100,$$
4. F1 Score: The F1 score is the harmonic average between the precision and the recall. The F1 score is given by Equation (16):
  (16) $$F1\;Score=2\;\times \;\frac{Precision\;\times \;Recall}{Precision+Recall}\times 100,$$
5. False Negative Rate: This is also known as the missed detection rate. It the probability that a true positive will be missed by the test.
  (17) $$False\;Negative\;Rate=\frac{False\;Negatives}{True\;Positives+False\;Positives}\times 100,$$
6. False Positive Rate: The false positive rate or the false alarm rate is expressed in Equation (18).
  (18) $$False\;Positive\;Rate=\frac{False\;Positives}{True\;Negatives+False\;Positives}\times 100,$$

## 4. Results and Discussion

### 4.1. Results from Individual Network

**Figure 3:**
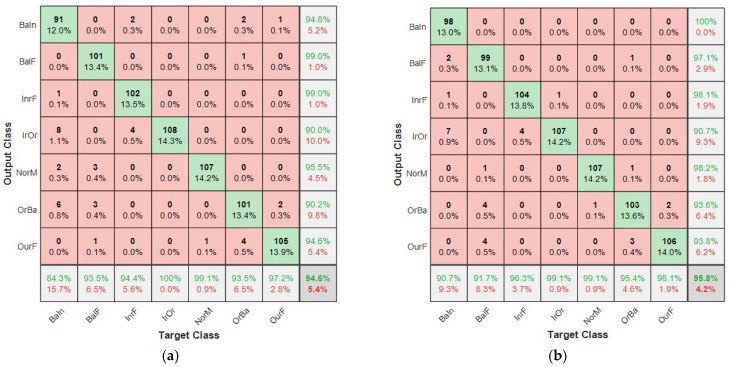
Confusion matrixes of: (**a**) CNN-1 model; (**b**) CNN-2 model.

The results from the individual learners indicate varied performance from the different tier-zero classifiers. The validation accuracy of these were 96.83%, 96.16% and 93.52%, for CNN-1, CNN-2, CNN-3, respectively. Taking a representative confusion matrix, as shown in Figure 3a, the rows represents the output class or predicted class while the column is the target class. The bottom row of the same figure shows the true positive rate and the false negative rate. However, the column at the far right indicates the precision and the false positive rate.

CNN-1 had a test accuracy of 94.60% on the 756 test dataset. It achieved a recall of 0.9458, precision of 0.9474 and F1 score of 0.9467 on the test dataset. Considering the NorM class in Figure 3a, the false positive rate was 4.5%, while the false negative rate was 0.9%. CNN–2 showed good modeling of inherent complex correlation in the dataset, with an overall test accuracy of 95.80%, as shown in the bottom right cell of Figure 3b. From Table 4, it is noticed that the amount of positive predicted value was 0.9592. The actual positive in the dataset, otherwise known as the sensitivity, was estimated to be 0.9577, while the F1 score for this classifier was 0.9584. Class NorM was noticed to have a false alarm rate of 1.8%, while the false negative rate was 0.9%.

**Figure 4:**
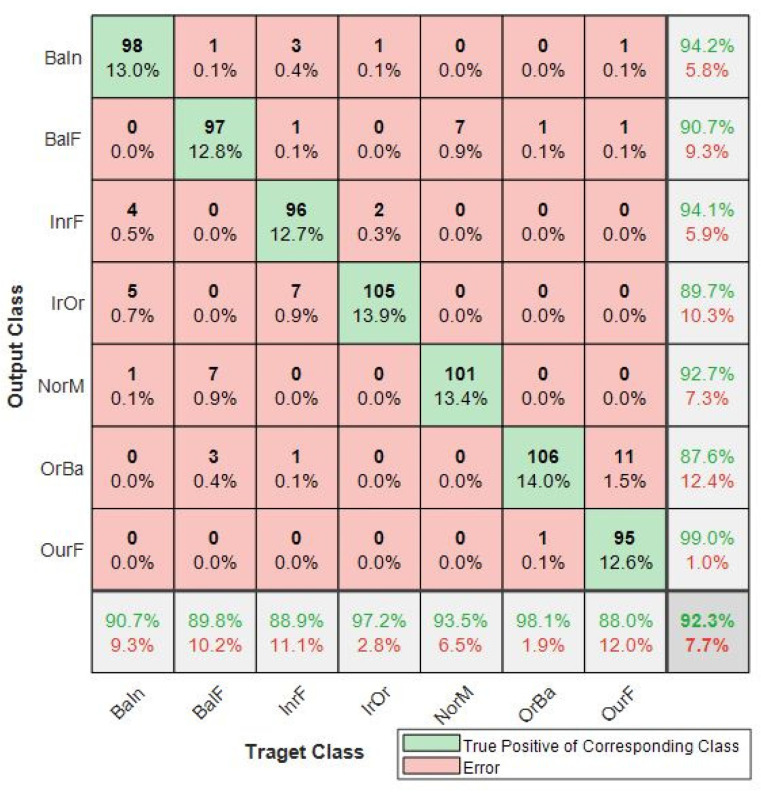
Confusion matrixes of CNN-3 model.

The CNN-3, bicoherence-based model, had an F1 score of 0.9245, precision of 0.9257, recall of 0.9233 and a test accuracy of 92.33%. The confusion matrix for this model in Figure 4 showed that the NorM class had a false positive rate of 7.3% and a false negative rate of 6.5%. Hierarchically, the CNN-2 performed better than CNN-1 and CNN-3 models. The results presented in Table 4 show that the proposed method has the highest accuracy.

### 4.2. Results from Ensemble Learning Methods

A common method for combining base models is averaging. Averaging has the advantage of reducing variance in the predictions based on the understanding that the based models will not make similar errors in their predictions [57]. It involves the generation and training of a specific number of models separately and combining them through the computation of the mean of the predicted class scores. The predicted class scores can be represented by a matrix “*N × K*”, where “*N*” is the number of samples and “*K*” is the number of classes. That is, the average from the predicted class scores of the three classifiers are estimated and the input pattern is assigned to the class with the maximum score among this mean [58]. The simple averaging is given mathematically by Equation (19). A comparison of the overall accuracy of the individual models and averaging in Table 4 shows a 2.20% increase in the latter’s accuracy and the best of the individual model.

(19) $$\mu_{c}\left(y\right)=\left(\frac{1}{T}\overset{T}{\underset{t}{\sum }}d_{t,c}\left(y\right)\right)\;,$$

where 1/*T* is the normalization factor, $\mu_{c}\left(y\right)$ is the maximum of total predicted class scores, $d_{t,c}\left(y\right)$ predicted score of individual classifiers to a class.

**Figure 5:**
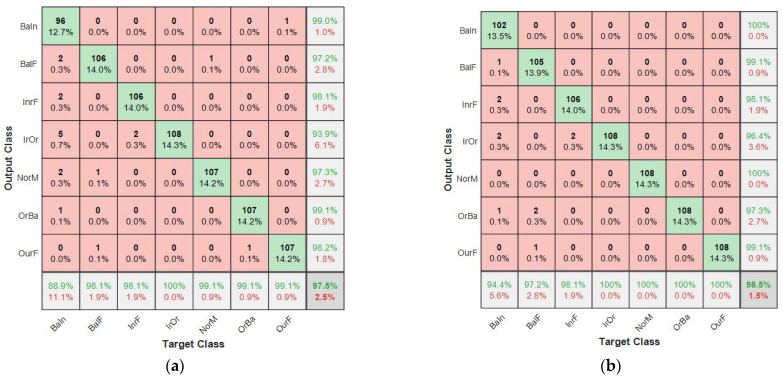
Confusion matrix for: (**a**) ECNN-DT; (**b**) proposed model (ECNN-SVM).

To study the effect of training with a different tier-one algorithm (meta-learner) on the overall results, the decision trees were introduced in the architecture as an alternative to the SVM. Decision trees are a well-known method and are fast to train [42]. The result from this modified blended ensemble model (ECNN-DT) was also better than the individual base models. However, it was noted that this approach for the representative class NorM produced 2.7% false positives and 0.9% false negatives. These rates were higher when compared with the proposed method. Figure 5a shows further details of the ECNN-DT and the proposed method.

Deep learning approaches have been proposed by other researchers for multiple faults diagnosis of rotating machines. The overall effectiveness of solutions by these authors using a multiple fault dataset are compared with our results and presented in Table 5. It indicates that overall, the proposed methods performed better.

### 4.3. Discusion

Multiple faults diagnosis of bearings is a challenging task. Hence, the development of a diagnostic system for reliable decision making is important. Such systems save resources by reducing downtime, missed detection and false alarms. In this article, CNNs, which constitutes the base learners, are used in a blending ensemble learning strategy for fault detection. A key aim of our approach is to exploit complementary preprocessing methods, the blending ensemble learning strategy and deep learning approach in bearing diagnostics. The blending ensemble learning strategy helps to improve the effectiveness of the overall model.

#### 4.3.1. Effect of Preprocessing Approaches

The choice of parameter for the preprocessing methods are of great importance. This is reflected in part through the overall performance of each of the networks. Selecting the appropriate segment size is vital to the success of the bicoherence estimation and the subsequent models. The level of decomposition chosen for the spectral kurtosis affects the accuracy of the CNN-1 model. Experimental observations have shown that the maximum level of decomposition provides a better result for the kurtogram-based CNN model. Due to the computation time required in creating the cyclic spectral coherence maps and the demand for high accuracy, the choice of the frame size is important. The frame size is indirectly proportional to cyclic frequency resolution. A small frame size will result in a poor resolution and contribute to reducing the performance of the model.

It is observed that the CNN-3 had the highest false positive and false negative rates. This can be attributed to the weakness of this preprocessing method. With the complex spectral components present in the multiple fault signal, the bicoherence does not present the cross correlation between the complex order spectral components of this type of signal [15].

#### 4.3.2. Discussions on the Ensemble and Individual Approaches

The models were evaluated by conducting experiments and comparing the results of the based learners independently. Further results from strategies such as averaging, ECNN-DT, and some previous works on multiple faults are compared based on the false positive rate and the false negative rate. In the experiments, data augmentation methods such as rotation, the range of horizontal translation, and the range of vertical translation, were implemented. The augmented training set was used to train the different individual CNN models.

Different performance metrics are presented in Table 4 and in Figure 5 for use in ascertaining the performance of the proposed diagnosis system. To maximize the gains of the health monitoring system, it is important to develop a model that minimizes both the false positive rates and the false negative rates. That way, the healthy or NorM bearings will not be replaced due to the diagnostic system wrongly indicating that it is faulty. Additionally, truly faulty component(s) will not be missed by the system.

A comparison of the confusion matrix for each of the independent CNN models that constitutes the base learners indicates a false positive rate for the NorM class as 0.9%, 0.9% and 6.5% for CNN-1, CNN-2, and CNN-3, respectively. This shows that out of the 108 test samples that made up the actual NorM bearings condition, CNN-1 presented 0.9% as having an outer race faults in Bearing 1, while CNN-2 showed 0.9% of the actual NorM bearing conditions as having outer race fault in Bearing 1 and inner race fault in Bearing 2. There was an increase in the false positive rate for CNN-3. The actual NorM bearing condition in this case was incorrectly classified. The false negative rates of CNN-1, CNN-2, and CNN-3 were recorded as 4.5%, 1.8% and 7.3%, respectively. The implication is, if the individual models are deployed individually for multiple fault diagnosis of bearings, a good amount of funds will be wasted in replacing actual good bearings.

The blended ensemble learning results are shown in Figure 5b. It is noticed that for the proposed model (ECNN-SVM), the false positive rate and false negative rate for NorM condition was 0%. This means that in all the test samples, none of the NorM bearing conditions were wrongly classified as faulty. Equally, none of the faulty bearing conditions were missed detected to be NorM. Hence, this increases the confidence of the diagnostic system. The results in Figure 5a show that the choice of the blender is also important to the success of this methods.

## 5. Conclusions

In this article, multiple fault diagnosis was conducted using vibration signal. The proposed solution was based on extended features achieved through three preprocessing methods. The models were fused in a homogenous blended ensemble learning method. This helped to effectively map information obtained in the feature space to the bearing fault space. When compared with individual models, the proposed method achieved better results. Hence, the overall effectiveness of the proposed method increased by 2.74% when compared to the best individual model. The results from this method showed 0% missed detection rate, 0% false alarm rate for the NorM class and 0.45% increase in overall effectiveness, compared with contemporary multiple fault diagnostic methods. An impact of this is that valuable resources will not be wasted in changing components which are not faulty and could contribute to eliminating catastrophic failures.

Using different pre-processing techniques is expected to improve the flexibility of the framework to new faults that will be projected into the proposed feature spaces and are expected to be detected by the CNN. Although this algorithm has not been tested directly with a complex systems dataset, results from the literature have indicated that cyclic spectral analysis, when used alone for fault isolation, showed good results for complex systems [50,62]. Future work is planned for this framework to be tested on more complex cases such as eccentric shafts, and the application of this method to uncontrolled environments.

## Figures and Tables

**Table 1:** Fault classes for the bearing.

S/No.	Fault Class	Bearing 1	Bearing 2
1	NorM	Normal	Normal
2	InrF	Inner race fault	Normal
3	OurF	Outer race fault	Normal
4	BalF	Ball fault	Normal
5	IrOr	Inner race fault	Outer race fault
6	BaIn	Inner race fault	Ball fault
7	OrBa	Outer race fault	Ball fault

**Table 2:** Composition of the training, validation, and test set.

Model	CNN-1	CNN-2	CNN-3	Run **
Training *	342	342	342	1 and 2
Validation	108	108	108	1 and 2
Testing	108	108	108	3

* Data augmentation was used, ** run of the machine.

**Table 3:** Structure of the tier-zero models.

Layer	Description	CNN-1	CNN-2	CNN-3
1	Input	224 × 224 × 3	224 × 224 × 3	224 × 224 × 3
2	conv_1	8 × 3 × 3 × 3	8 × 5 × 5 × 3	16 × 5 × 5 × 3
3	maxpool_1	3 × 3	3 × 3	3 × 3
4	conv_2	16 × 3 × 3 × 8	16 × 5 × 5 × 8	16 × 5 × 5 × 16
5	maxpool_2	3 × 3	3 × 3	3 × 3
6	conv_3	32 × 3 × 3 × 16	32 × 5 × 5 × 16	32 × 5 × 5 × 16
7	maxpool_3	3 × 3	3 × 3	3 × 3
8	conv_4	64 × 3 × 3 × 32	64 × 5 × 5 × 32	64 × 5 × 5 × 32
9	dropout	10%	30%	10%
10	fully Connected	fully Connected	fully Connected	fully Connected
11	SoftMax	1	1	1
12	class output	7	7	7

**Table 4:** Performance of the models.

Model	Test Accuracy (%)	Precision (%)	Recall (%)	F1 Score
CNN-1	94.60	94.74	94.58	0.9466
CNN-2	95.80	95.92	95.77	0.9584
CNN-3	92.33	92.57	92.33	0.9245
Averaging	98.02	98.10	98.02	0.9806
ECNN-DT	97.50	97.54	97.49	0.9751
**Proposed Method**	**98.54**	**98.57**	**98.54**	**0.9855**

**Table 5:** Performance comparison with other deep learning approaches.

Authors	Approach	Rotating Components Used	Results Accuracy (%)
Han et al. [33]	Multi-level wavelet packet fusion in dynamic CNN	Bearings and Gear	96.48 (Case 1) 91.30 (Case 2)
Lu et al. [24]	Hierarchical CNN	Bearing	92.60
Ma et al. [34]	Ensemble deep-learning	Rotor and Bearing faults	98.09
Yu et al. [59]	Autoencoders	Gear and Bearings	95.50 (Avg.)
Li et al. [32]	EWV + thresholds + BAS	Bearings	96.92
Shao et al. [60]	Deep autoencoder feature learning	Bearing and Gear	87.80
Sri et al. [61]	Multiple Classifiers and Data Fusion/CWT/CNN	Mixed gearbox fault	98.0
**Proposed Method**	**Proposed Method**	**Multiple faults bearings**	**98.54**

## Data Availability

Not applicable.
